# Salvage treatment for recurrences after first resection of colorectal liver metastases: the impact of histopathological growth patterns

**DOI:** 10.1007/s10585-019-09960-7

**Published:** 2019-03-06

**Authors:** Pieter M. H. Nierop, Boris Galjart, Diederik J. Höppener, Eric P. van der Stok, Robert R. J. Coebergh van den Braak, Peter B. Vermeulen, Dirk J. Grünhagen, Cornelis Verhoef

**Affiliations:** 1 Department of Surgical Oncology and Gastrointestinal Surgery, Erasmus MC Cancer Institute, P.O. Box 2040, 3000 CA Rotterdam, The Netherlands; 2 Department of Surgery, Erasmus Medical Centre, Rotterdam, The Netherlands; 3 Translational Cancer Research Unit, (GZA Hospitals and University of Antwerp), Antwerp, Belgium

**Keywords:** Colorectal liver metastases, Histopathological growth patterns, Salvageable recurrences, Biomarker, Prognostication

## Abstract

The majority of patients recur after resection of colorectal liver metastases (CRLM). Patients with CRLM displaying a desmoplastic histopathological growth pattern (dHGP) have a better prognosis and lower probability of recurrence than patients with non-dHGP CRLM. The current study evaluates the impact of HGP type on the pattern and treatment of recurrences after first resection of CRLM. A retrospective cohort study was performed, including patients with known HGP type after complete resection of CRLM. All patients were treated between 2000 and 2015. The HGP was determined on the CRLM resected at first partial hepatectomy. The prognostic value of HGPs, in terms of survival outcome, in the current patient cohort were previously published. In total 690 patients were included, of which 492 (71%) developed recurrent disease. CRLM displaying dHGP were observed in 103 patients (21%). Amongst patients with dHGP CRLM diagnosed with recurrent disease, more liver-limited recurrences were seen (43% vs. 31%, p = 0.030), whereas patients with non-dHGP more often recurred at multiple locations (34% vs. 19%, p = 0.005). Patients with dHGP CRLM were more likely to undergo curatively intended local treatment for recurrent disease (adjusted odds ratio: 2.37; 95% confidence interval (CI) [1.46–3.84]; p < 0.001) compared to patients with non-dHGP. The present study demonstrates that liver-limited disease recurrence after complete resection of CRLM is more often seen in patients with dHGP, whereas patients with non-dHGP more frequently experience multi-organ recurrence. Recurrences in patients with dHGP at first CRLM resection are more likely to be salvageable by local treatment modalities, but no prognostic impact of HGPs after salvage therapy for recurrent disease was found.

## Introduction

After hepatic resection for colorectal liver metastases (CRLM) the majority of patients experiences recurrence of disease. Despite advances in the treatment of CRLM, recurrence rates reach up to 70% [1–5]. Approximately 40% of the patients with recurrent disease is again eligible for local treatment modalities [4, 6–8]. If disease biology allows the recurrence to be treated locally again, survival outcomes similar to the first local treatment of metastases are seen [1, 4, 6–13]. In case of a recurrence not amenable to local treatment prognosis is limited [4, 7, 8, 13]. In addition, clinical risk factors currently used for the prediction of prognosis after first hepatic resection for CRLM, have not proven equally useful in prognostication after repeat resection for recurrent CLRM [14].

Histopathological growth patterns (HGPs) describe the transition border of CRLM to the normal liver parenchyma [15]. The assessment of HGPs has been standardised in international consensus guidelines [16] and multiple studies have reported the effect of HGPs on prognosis in patients with resectable CRLM [16–22]. We recently described the largest patient cohort to date and found that the desmoplastic HGP (dHGP) is associated with favourable overall survival, progression free survival compared to its non-desmoplastic counterpart (non-dHGP) [23]. In the current study we aimed to identify in the same cohort of patients potential explanations for this survival difference. Differences in recurrence pattern (intra- versus extrahepatic) and/or treatment of recurrent disease (local vs. systemic) might possibly account for the difference in survival outcomes between HGPs. Therefore, the current study investigates the pattern of first recurrence and the salvageability of recurrent disease after first partial hepatectomy for CRLM in the context of HGPs.

## Methods

### Patients

The current study was approved by the medical ethics committee of the Erasmus University Medical Centre Rotterdam (MEC 2018-1743). All consecutive patients that underwent first surgical treatment for CRLM between 2000 and 2015 at the Erasmus MC Cancer Institute were considered for inclusion. The prognostic value of HGPs, in terms of survival outcome, in the current patient cohort were previously published [23]. Patients selected for this study had to be completely free of all known macroscopic disease at some point following first resection of CRLM in order to be eligible for inclusion. A positive resection margin (R1) was defined as tumour cells (i.e. microscopic residual disease) at the resection margin and therefore patients with an R1 resection were eligible for inclusion. Patients with unknown HGP type were excluded.

### Design and outcomes

Data on patient characteristics, primary tumour, CRLM and recurrence were extracted from a prospectively maintained database. H&E tissue sections were retrospectively analysed for HGP assessment. Disease free survival (DFS) was defined as the time in months between the first hepatic resection for CRLM and diagnosis of recurrence or death. Post-recurrence survival (PRS) was defined as the time in months between diagnosis of recurrence after first hepatic resection for CRLM and death. When alive patients were censored at date of last follow-up. Local therapy with curative intent was defined as resection, ablation and/or radiation therapy after which the patient was considered to be free of disease.

### Treatment and follow-up after first partial hepatectomy

Perioperative chemotherapy for resectable CRLM is not standard of care in the Netherlands, since no OS benefit has been found in randomised setting [24]. Therefore preoperative chemotherapy at the Erasmus MC Cancer Institute is only considered in case of borderline resectable, more than four and/or synchronous CRLM. Some patients, however, received chemotherapy in referring hospitals prior to referral. Patients do not receive postoperative chemotherapy. Follow-up is performed up to 5 years after resection of CLRM. The follow-up consists of carcinoembryonic antigen (CEA) monitoring every 3 months for the entire follow-up duration and imaging every 6 months in the first 3 years and annually in the fourth and fifth year. In case of elevated CEA levels (> 5 µg/L) or a rise in CEA levels (> 25%) imaging is performed. When uncertainty with regard to the diagnosis of disease recurrence exists, biopsies are taken as confirmation. As with primary treatment for CRLM, treatment strategy for recurrent disease is established by a multidisciplinary board. The decision whether local therapies (resection, ablation, stereotactic body radiation) are considered beneficial for patients, depends on two factors: time to recurrence and localisation of recurrences.

Regarding time to recurrence, it was previously demonstrated that patients with a disease-free interval of less than 6 months again undergoing local treatment for the recurrence have poor survival outcomes [25]. Therefore, when patients present with recurrent disease within 6 months after resection of CRLM, patients first receive systemic chemotherapy before local therapy is considered. Systemic therapy normally consists of oxaliplatin- or irinotecan-based treatment regimens. Typically, three courses are administered followed by restaging and local therapy in case of partial response or stable disease. In case of progressive disease, patients are switched to second line chemotherapeutic regimens. When patients present with recurrent disease beyond 6 months after primary liver resection for CRLM and the lesions are treatable with local therapy, these patients are planned for local therapy accordingly. Again, no adjuvant chemotherapy is administered. Patients presenting with recurrent disease not eligible for local treatment receive palliative treatment.

Provided that the interval between first liver resection and recurrence is greater than 6 months, or less than 6 months, but at least stable disease after three courses of chemotherapy is observed, then localisation of recurrences is a decisive factor in the clinical decision making in these patients. The currently handled standard at our centre is, that when recurrent disease is liver-limited and it can be resected with sufficient remnant liver, local treatment of the colorectal liver metastases should be attempted. In addition, local treatment is deemed feasible when concurrent oligometastatic extrahepatic is present. When extrahepatic disease is present in > 1 organ, local treatment is deemed futile.

### HGP assessment

The HGPs were determined on the CRLM resected at the first hepatectomy. The HGP of CRLM describes the tumour-liver interface. Three different types of HGPs have been described; the desmoplastic (dHGP), the replacement (rHGP) and the rare pushing HGP (pHGP) [16]. The latter two (rHGP and pHGP) can be taken together as non-dHGP, since recent findings indicate that patients with CRLM that display *any* proportion non-dHGP at the interface have impaired prognosis compared to patients with pure dHGP [23]. In this study, international consensus guidelines for HGP assessment of liver metastases were utilised to determine the HGPs [16]. HGP determination was jointly executed by at least three trained observers (PN, BG, DH, ES, RC, PV). The observers were blinded for clinical data and outcome during HGP assessment. Some CRLM display multiple HGPs, therefore the complete interface of all available H&E tissue sections of all CRLM in every patient were examined. Only if pure dHGP was observed, patients were categorised as such. All other patient displaying any non-dHGP were categorised as non-dHGP. In accordance with the consensus guidelines, not all tissue sections are suitable for HGP assessment. If less than 20% of the interface is assessable, if the tissue section is of insufficient quality or when no vital tumour is present, the HGP cannot be determined.

### Statistical analysis

Categorical data were presented using counts and percentages. Continuous data were reported with medians and corresponding interquartile range (IQR). Differences in proportions were evaluated with the Chi-squared test. Medians were compared using the Mann–Whitney U test. Median follow-up time for survivors was estimated by means of the reversed Kaplan–Meier method. Survival estimates were obtained using the Kaplan–Meier method, computed until 60 months and compared with the log rank test. Uni- and multivariable Cox regression analysis was performed to correct for potential confounding. Results of the Cox regression analyses were expressed in hazard ratios (HR) and corresponding 95% confidence intervals (CI). Uni- and multivariable binary logistic regression analysis was performed to evaluate possible predictors for unsalvageable recurrence. Results of the logistic regression analyses were expressed in odds ratios (OR) and corresponding 95% CI. In both the binary logistic regression and the Cox univariable regression models, all variables potentially related to salvageability of recurrence and/or overall survival were considered. All variables with p-values < 0.100 on univariable analysis were entered in the multivariable models. All statistical tests were two-sided and p-values < 0.05 were considered statistically significant. All analyses were performed using SPSS version 24.0 (SPSS Inc., Chicago, IL) and R version 3.5.1 (http://www.r-project.org).

## Results

### Patients and disease free survival

During the study period 964 patients were treated surgically for CRLM at the Erasmus MC Cancer Institute. HGP determination was performed in 732 patients (76%). Patients were excluded due to: no (complete) resection of CRLM (n = 100), missing H&E tissue sections (n = 55), ablative therapy only (n = 21) or non-suitable H&E tissue sections for HGP determination (n = 56). Of these 732 patients, 690 were completely free of all known disease at some point following first resection of CRLM and were included in the study. Hence, 42 patients were excluded (n = 24 primary tumour never resected after liver-first approach due to progressive metastatic disease, n = 18 extrahepatic disease never treated locally).

**Fig. 1:**
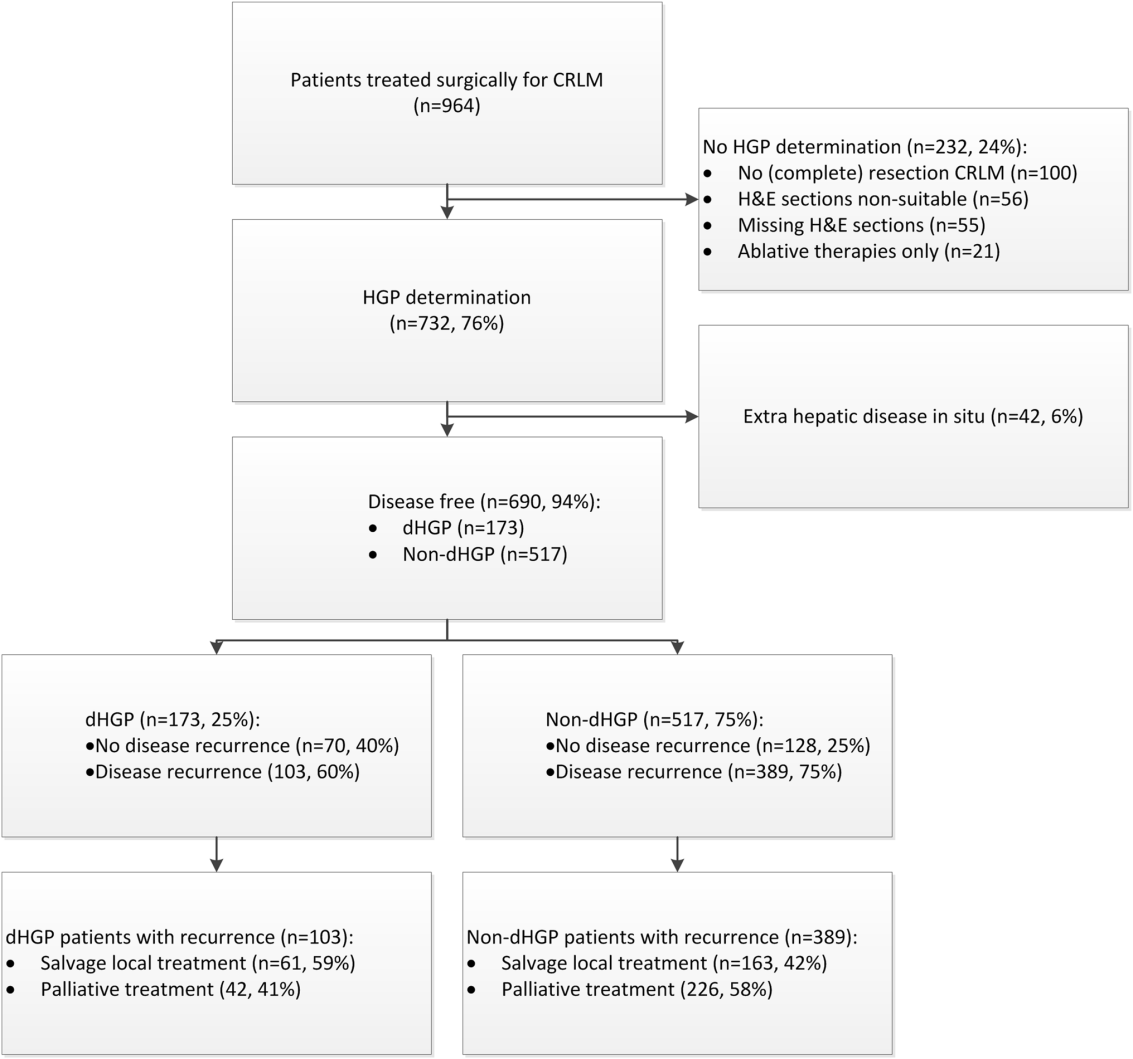
Flowchart of patient selection

Among the included patients, there were 173 (25%) with dHGP and 517 with non-dHGP (75%). Median follow-up for survivors was 76 months (IQR: 45–116). In total 492 patients (71%) had disease recurrence. A flowchart of the patient inclusion is displayed in Fig. 1. Baseline characteristics of all 690 patients compared for HGP are reported in Table 1. At baseline there were several differences between patients with dHGP compared to patients non-dHGP, especially in terms of primary tumour characteristics (lymph node status and adjuvant treatment) and CRLM characteristics (disease-free interval, CEA, size of largest CRLM, resection margin and preoperative treatment).

**Table 1:** Baseline characteristics of all patients stratified for HGP

	Total N = 690	dHGP N = 173	Non-dHGP N = 517	p-value
Gender				
Male	445 (65%)	109 (63%)	336 (65%)	0.637
Female	245 (36%)	64 (37%)	181 (35%)	
Age				
Median (IQR)	65 (58–71)	65 (56–72)	64 (58–71)	0.984
ASA				
ASA I-II	617 (91%)	153 (89%)	464 (91%)	0.351
ASA > II	63 (9%)	19 (11%)	44 (9%)	
Missing	10 patients			
Primary tumour characteristics				
Location				
Right-sided	116 (17%)	30 (17%)	86 (17%)	0.927
Left-sided	302 (44%)	76 (44%)	226 (44%)	
Rectum	256 (37%)	62 (36%)	194 (38%)	
Double tumour	16 (2%)	5 (3%)	11 (2%)	
pTumour stage				
pT0-2	134 (20%)	39 (23%)	95 (19%)	0.239
pT3-4	546 (80%)	132 (77%)	414 (81%)	
Missing	10 patients			
Nodal status				
N0	270 (40%)	79 (47%)	191 (38%)	0.035*
N+	407 (60%)	90 (53%)	317 (62%)	
Missing	13 patients			
Adjuvant chemotherapy primary tumour				
No	587 (85%)	160 (93%)	427 (83%)	0.002*
Yes	103 (15%)	13 (8%)	90 (17%)	
CRLM characteristics				
Synchronous CRLM				
No	329 (48%)	264 (51%)	65 (38%)	0.002*
Yes	361 (52%)	253 (49%)	108 (62%)	
Disease-free interval (months)				
Median (IQR)	2 (0–17)	0 (0–13)	5 (0–18)	0.006*
Number of CRLM				
Median (IQR)	2 (1–4)	2 (1–4)	2 (1–4)	0.886
Size of largest CRLM (cm)				
Median (IQR)	3.1 (2.0-4.5)	2.5 (1.8–4.2)	3.3 (2.3–4.8)	< 0.001*
Missing	2 patients			
Preoperative CEA (µg/L)				
Median (IQR)	14.0 (4.7–50.0)	7.6 (3.2–30.0)	16.2 (5.1–53.0)	< 0.001*
Missing	28 patients			
Fong CRS				
Low	408 (61%)	101 (61%)	307 (61%)	0.924
High	262 (39%)	64 (39%)	198 (39%)	
Incomplete CRS	20 patients			
Bilobar metastases				
No	418 (61%)	106 (61%)	312 (60%)	0.830
Yes	272 (39%)	67 (39%)	205 (40%)	
Preoperative CTx				
No	365 (53%)	68 (39%)	297 (57%)	< 0.001*
Yes	325 (47%)	105 (61%)	220 (43%)	
Resection margin				
R0	585 (85%)	158 (92%)	427 (83%)	0.004*
R1	102 (15%)	14 (8%)	88 (17%)	
Missing	3 patients			
Extra hepatic disease				
No	629 (91%)	157 (91%)	472 (91%)	0.827
Yes	61 (9%)	16 (9%)	45 (9%)	
Major liver resection				
<3 complete segments	455 (66%)	122 (71%)	333 (64%)	0.142
≥3 complete segments	235 (34%)	51 (30%)	184 (36%)	
Recurrence after first resection CRLM				
No	198 (29%)	70 (40%)	128 (25%)	< 0.001*
Yes	492 (71%)	103 (60%)	389 (75%)	

*Indicates significant p-value

Percentages do not always add up to 100% due to rounding

*ASA* American Society of Anaesthesiologists, *CEA* carcinoembryonic antigen, *CRLM* colorectal liver metastases, *CRS* clinical risk score, *CTx* Chemotherapy, *HGP* histopathological growth pattern, *IQR* interquartile range, *R1* irradical resection margin

### Recurrence: survival, pattern and treatment

**Fig. 2:**
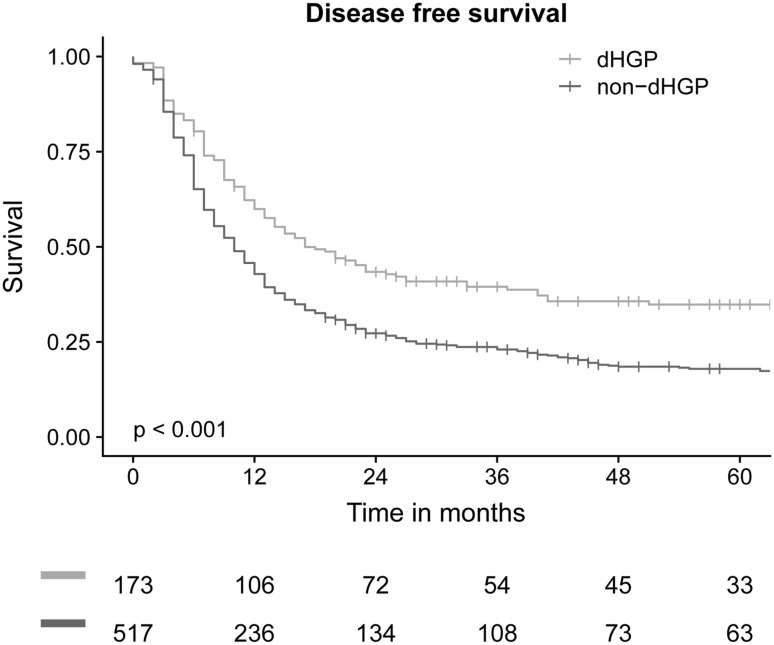
DFS after first hepatic resection for CRLM compared for HGP

A smaller proportion of patients with dHGP had disease recurrence compared to patients with non-dHGP (60% vs. 75%). Median DFS of patients with dHGP was 17 months (IQR: 7-not reached) compared to 10 months (IQR: 5–28) in patients with non-dHGP. The DFS significantly differed between both groups (p < 0.001, Fig. 2).

In total 492 patients had disease recurrence after first resection of CRLM. The median time to recurrence in these 492 patients with recurrent disease was 8 months (IQR: 5–14). This was 9 months (IQR: 6–14) in patients with dHGP compared to 8 months (IQR: 4–13 months) in patients with non-dHGP. At 6 months after first liver resection, 57% of patients with non-dHGP developing recurrences was disease-free, while 71% of patients with dHGP tumours developing recurrences was disease-free at this point in time. Data on the pattern of first recurrence stratified for HGP are reported in Table 2. Patients with dHGP at first partial hepatectomy more often had an intrahepatic only recurrence (43% vs 31%, p = 0.030) whereas patients with non-dHGP more often had a multi-organ (≥ 2) recurrence (34% vs 19%, p = 0.005). Of all 492 patients with a recurrence, 224 (46%) were again treated with curative intent. Patients with dHGP were more often treated with curative intent for the recurrence (59% vs. 42%, p = 0.002). After correction for potential confounders, dHGP at first partial hepatectomy remained a significant predictor for salvageable recurrence (OR: 2.37, p < 0.001). Significant predictors negatively associated with salvageability were a right-sided primary tumour (OR: 0.36, p < 0.001), a node positive primary tumour (OR: 0.57, p = 0.008) and larger CRLM at first partial hepatectomy (OR: 0.92, p = 0.036) (Table 3).

**Table 2:** Recurrence pattern

	Total (N = 492)	dHGP (N = 103)	Non-dHGP (N = 389)	p-value
Intrahepatic only	166 (34%)	44 (43%)	122 (31%)	0.030*
Pulmonary only	104 (21%)	22 (21%)	82 (21%)	0.951
One other location only	70 (14%)	17 (17%)	53 (14%)	0.457
Local recurrence primary only	15 (3%)	3 (3%)	12 (3%)	
Peritoneal only	3 (1%)	1 (1%)	2 (1%)	
Distant lymph nodes only	26 (5%)	7 (7%)	19 (5%)	
Other location only	26 (5%)	6 (6%)	20 (5%)	
Two or more locations	152 (31%)	20 (19%)	132 (34%)	0.005*
Intrahepatic and pulmonary only	49 (10%)	10 (10%)	39 (10%)	
Intrahepatic and 1 other only	41 (8%)	3 (3%)	38 (10%)	
Pulmonary and 1 other only	25 (5%)	1 (1%)	24 (6%)	
Peritoneal and 1 other only	2 (1%)	0 (0%)	2 (1%)	
Multi organ (> 2)	35 (7%)	6 (6%)	29 (8%)	
Treatment of recurrence with curative intent	224 (46%)	61 (59%)	163 (42%)	0.002*

*Indicates significant p-value

**Table 3:** Logistic regression for salvageable recurrence

Variable	Univariable		Multivariable	
	Odds ratio [95% CI]	P-value	Odds ratio [95% CI]	p-value
Age at resection CRLM (cont.)	0.986 [0.968–1.004]	0.122		
ASA > II	0.879 [0.470–1.642]	0.685		
Right-sided primary	0.416 [0.249–0.694]	0.001*	0.364 [0.211–0.628]	< 0.001*
pT3-4	0.534 [0.334–0.855]	0.009*	0.686 [0.409–1.151]	0.153
Node positive primary	0.490 [0.336–0.715]	< 0.001*	0.568 [0.375–0.860]	0.008*
Disease free interval (cont.)	1.011 [1.001–1.022]	0.037*	1.013 [1.003–1.024]	0.014*
Number of CRLM (cont.)	0.949 [0.880–1.023]	0.171		
Diameter largest CRLM (cont.)	0.932 [0.862–1.007]	0.076	0.915 [0.842–0.994]	0.036*
Preoperative CEA level (cont.)	1.000 [0.999-1.000]	0.270		
Preoperative chemotherapy	1.210 [0.849–1.727]	0.292		
R1 resection CRLM	0.971 [0.607–1.554]	0.903		
Extra hepatic disease	0.864 [0.483–1.545]	0.622		
Desmoplastic type tumours	2.014 [1.295–3.132]	0.002*	2.370 [1.462–3.840]	< 0.001*

*Indicates significant p-value

*ASA* American Society of Anaesthesiologists, *CEA* carcinoembryonic antigen, *cont*. continuous, *CRLM* colorectal liver metastases, *R1* irradical resection margin

As the higher rate of intrahepatic only recurrences in the dHGP group might explain the higher likelihood of curatively intended salvage treatment additional analyses have been performed, specifically excluding patients with intrahepatic recurrences only. We subsequently conducted the same multivariable logistic regression analysis as conducted previously and, despite excluding patients with liver-limited recurrences, still found a statistically significant association between dHGP and salvage treatment of the recurrence (adjusted OR: 3.16, p < 0.001).

### Post-recurrence survival

**Fig. 3:**
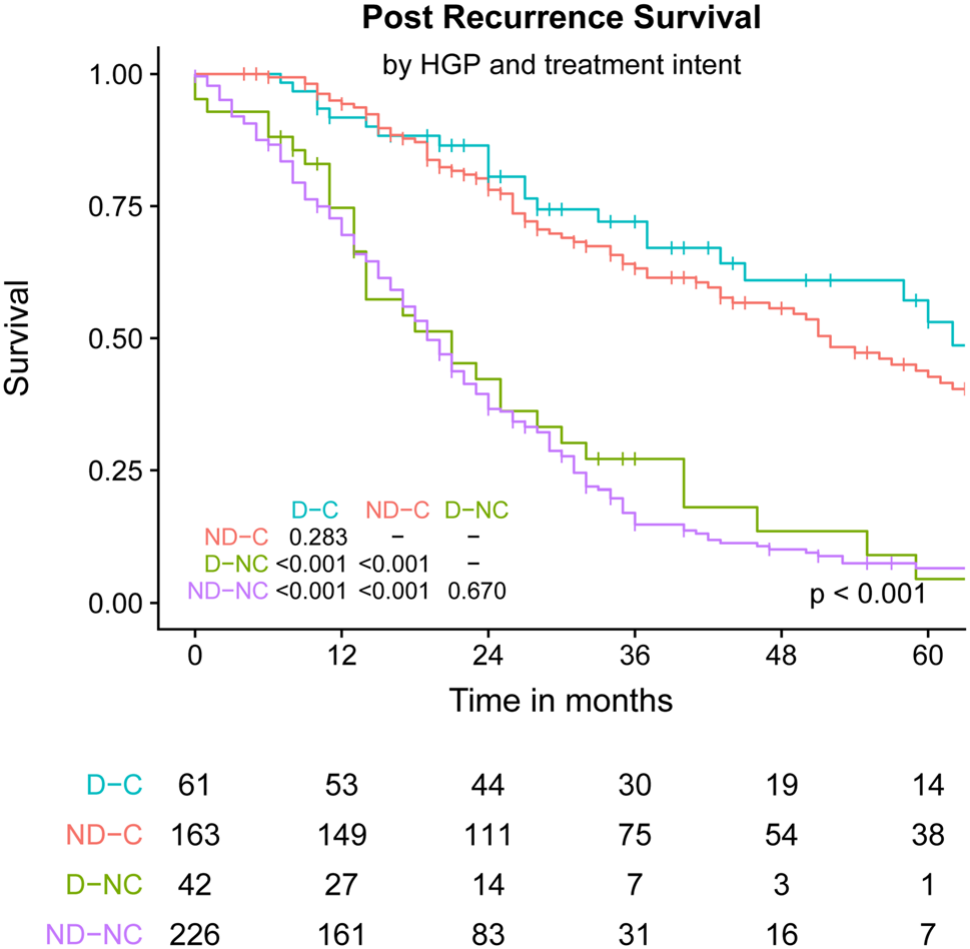
PRS compared for HGP and treatment intent of the recurrence. *D-C* dHGP and curative intent, *ND-C* Non-dHGP and curative intent, *D-NC* dHGP and non-curative intent, *ND-NC* Non-dHGP and non-curative intent

Median PRS after diagnosis of recurrence was 28 months (IQR: 15–59 months). Patients treated with curative intent had a median PRS of 56 months (IQR: 27–84 months) compared to 19 months (IQR: 11–32 months) for patients receiving palliative treatment (p < 0.001). After stratification for treatment intent, no difference in PRS was observed between patients with dHGP and non-dHGP (both p-values > 0.25, Fig. 3).

## Discussion

The current study demonstrates that patients with dHGP at first CRLM resection more often develop an intrahepatic only recurrence, whereas patients with non-dHGP more often experience multi-organ recurrence. Importantly, dHGP at first CRLM resection is independently associated with salvageable recurrences after first partial hepatectomy for CRLM. Prognosis after salvage treatment for recurrent disease is not impacted by HGP type determined at first resection of CRLM.

Unfortunately, the majority of patients develops a recurrence after curatively intended resection of CRLM [1–12]. The prognosis of patients with recurrent disease strongly depends on whether local treatment can still be performed. Disease load and tumour biology largely determine if local therapy is possible and beneficial [4, 10, 12, 26]. As this study shows, that recurrences in patients with dHGP at first CRLM resection are more likely to be salvageable, this potentially explains the observed outcome difference between patients with dHGP and non-dHGP. Several studies have suggested that dHGP is associated with favourable tumour characteristics and a lower recurrence rate [16–23]. The more favourable tumour behaviour of dHGP CRLM was further acknowledged in this study, as patients with dHGP at first CRLM resection more often experience intra-hepatic only recurrence, whereas patients with non-dHGP more often develop multi-organ metastases. This also partially explains why salvage therapy was more often performed in these patients, as repeat resection of isolated recurrences is often feasible [1, 4, 6, 7, 9–12]. There were several differences observed at baseline between patients with dHGP compared to patients with non-dHGP in terms of clinical risk. Patients with non-dHGP had a greater proportion lymph node positive primaries, larger CRLM, and more often an R1 resection margin. These differences might also have attributed to the greater risk of multi-organ recurrences that are less likely salvageable with local treatment modalities in patients with non-dHGP. However, after correction for potentially confounding factors, dHGP remained significantly associated with salvageable recurrences. In addition, this study shows that patients with dHGP less often develop a recurrence and, if they do, the recurrence is also more often salvageable with local treatment modalities.

A frequently debated contraindication for local treatment of colorectal liver metastases is the simultaneous presence of extrahepatic disease. However, several recent (reviews of) retrospective series support resection of liver metastases and concurrent mono-organic extrahepatic disease in highly selected patients [27–30]. When extrahepatic disease is present in > 1 organ, the benefit of local treatment seems questionable as it holds outcome similar to systemic treatment alone [30]. As we demonstrated that multi-organ metastasis are more often found in patients with non-dHGP, we believe that this also partially explains why salvage treatment is less often performed in these patients. Moreover, several studies have demonstrated that some localisations of (recurrent) metastases (e.g. liver and concurrent para-aortic lymph node metastases [31, 32]) are associated with poor survival outcomes after surgery. Therefore, local therapies are often not considered beneficial in these patients. The true value of maximal tumour debulking in metastatic colorectal cancer will only be known after the completion of the ongoing ORCHESTRA trial (NCT01792934) in which patients are randomised between chemotherapy alone or the combination of chemotherapy and maximal tumour debulking.

The differences in recurrence patterns between HGP types might have implications for perioperative treatment. As patients with non-dHGP at first CRLM resection more often develop multi-organ recurrence, one could hypothesize that perioperative chemotherapy is more effective in these patients, since patients at high risk of (systemic) recurrence appear to benefit more from perioperative systemic treatment [33, 34]. Vice versa, patients with dHGP at first CRLM resection might benefit more from hepatic arterial infusion (HAI) chemotherapy as they are more likely to develop recurrences confined to the liver. This hypothesis is supported by the recent finding that patients with low clinical risk, and therefore are less likely to develop extrahepatic disease, appear to benefit the most from HAI chemotherapy whereas patients with extrahepatic disease do not seem to benefit from HAI chemotherapy [35]. Future studies should evaluate the effect of perioperative treatment in the context of HGPs.

As the scoring was performed jointly and the final HGP score was determined by consensus between all observers, no Kappa value for this specific study can be provided. However, in another recently submitted manuscript by our group we have found excellent Kappa indices (> 0.9) for discrimination between dHGP and non-dHGP [36].

This is the first paper demonstrating a significant association between distinct HGPs and differences in recurrence pattern in patients treated surgically for CRLM. Eefsen and colleagues [18] reported on the recurrence pattern in the context of HGPs but did not find an association. Importantly, the authors in that study applied an arbitrary cut-off value for the determination of the pre-dominant HGP. Recent insights have shown that the presence of any non-dHGP entails poor prognosis and no cut-off value for determination of the predominant HGP should be applied [23]. In addition, the number of patients with a recurrence in their study was limited and therefore a potential lack of power should also be considered. The current study handled no arbitrary cut-off value for pre-dominant HGP determination and describes a sufficiently large cohort, in which proper correction for confounding could be performed.

Most of the currently available risk factors for worse outcome after first resection of CRLM do not hold similar prognostic value when utilised for preoperative prognosis prediction at repeat resection of recurrent CRLM [14]. This indicates that there is a need for new prognostic markers in patients undergoing repeat partial hepatectomies for recurrent CRLM. This is the first study to evaluate the prognostic impact of HGPs of the CRLM resected at first liver resection for prognosis after repeat resection of CRLM. No difference in PRS was observed between patients with dHGP and non-dHGP. The reason that the HGP of the CRLM resected at first liver resection, rather than the HGP of recurrent CRLM resected at repeat resection, were used in the current study was twofold. Firstly, if the HGP at first resection had proven to be prognostic after repeat resection it would have become not only a predictive marker for prognosis after first resection, but also a pre-salvage treatment marker for local treatment of the recurrence. Secondly, this cohort also describes patients with an extrahepatic recurrence without a concurrent hepatic recurrence and therefore no HGP of an recurrent CRLM could be utilised.

Recently RAS mutational status has also been associated with unsalvageable recurrences [4]. Unfortunately RAS and BRAF mutational status were unknown in the currently described patient cohort at time of resection. In an attempt to correct for this drawback, primary tumour location (right- vs. left-sided) was taken into account in the multivariable analysis. Right-sided tumours have been associated with the presence of KRAS [37, 38] and BRAF [37–40] mutations. Right-sidedness of the primary tumour was independently negatively associated with salvageability of recurrent disease in the present study. Despite correcting for primary tumour location (and thereby partially correcting for mutational status) HGP type remained statistically associated with salvageability of recurrent disease.

The limitations of the current study should be taken into account. Although data was extracted from a prospectively maintained database, HGP determination was performed retrospectively. Also, in 96 potentially eligible patients no HGP could be determined, which might have induced selection bias. The prognostic value of HGPs and their association with salvageability of recurrent disease after first resection of CRLM should therefore be validated, preferably in a prospective setting.

In conclusion, the present study confirms that over two-thirds of patients develop a recurrence after primary resection of CRLM. Disease recurrence confined to the liver is more often seen in patients with dHGP at first CRLM resection whereas patients with non-dHGP more frequently develop multi-organ recurrence. Importantly, recurrences in patients with dHGP at first CRLM resection are more likely to be salvageable by local treatment modalities. HGPs determined at first CRLM resection had no prognostic value after salvage therapy for recurrent disease.
